# Modeling Intraday *Aedes*-human exposure dynamics enhances dengue risk prediction

**DOI:** 10.1038/s41598-025-91950-9

**Published:** 2025-03-07

**Authors:** Steffen Knoblauch, Julian Heidecke, Antônio A. de A. Rocha, Paulo Filemon Paolucci Pimenta, Marcel Reinmuth, Sven Lautenbach, Oliver J. Brady, Thomas Jänisch, Bernd Resch, Filip Biljecki, Joacim Rocklöv, Annelies Wilder-Smith, Till Bärnighausen, Alexander Zipf

**Affiliations:** 1 GIScience Research Group, Heidelberg University, Heidelberg, Germany; 2 Interdisciplinary Centre of Scientific Computing (IWR), Heidelberg University, Heidelberg, Germany; 3 HeiGIT at Heidelberg University, Heidelberg, Germany; 4 Institute of Computing, Fluminense Federal University (UFF), Niterói, Brazil; 5 Fundação Oswaldo Cruz, Instituto de Pesquisas René Rachou, Belo Horizonte, MG Brazil; 6 Centre for the Mathematical Modelling of Infectious Diseases, London School of Hygiene and Tropical Medicine, London, UK; 7 Department of Infectious Disease Epidemiology, Faculty of Epidemiology and Population Health, London School of Hygiene and Tropical Medicine, London, UK; 8 Centre on Climate Change and Planetary Health, London School of Hygiene and Tropical Medicine, London, UK; 9 Center for Global Health and Department of Epidemiology, Colorado School of Public Health, Aurora, USA; 10 Interdisciplinary Transformation University Austria, Linz, Austria; 11 Center for Geographic Analysis, Harvard University, Cambridge, USA; 12 Department of Architecture, National University of Singapore, Singapore, Singapore; 13 Department of Real Estate, National University of Singapore, Singapore, Singapore; 14 Heidelberg Institute of Global Health (HIGH), University Hospital Heidelberg, Heidelberg, Germany; 15 World Health Organization, Geneva, Switzerland

**Keywords:** Urban dengue transmission, Daytime exposure, *Aedes* biting rates, Human movement, Urban mobility, Spatial eigenvector mapping, Infectious diseases, Ecological epidemiology

## Abstract

Cities are the hot spots for global dengue transmission. The increasing availability of human movement data obtained from mobile devices presents a substantial opportunity to address this prevailing public health challenge. Leveraging mobile phone data to guide vector control can be relevant for numerous mosquito-borne diseases, where the influence of human commuting patterns impacts not only the dissemination of pathogens but also the daytime exposure to vectors. This study utilizes hourly mobile phone records of approximately 3 million urban residents and daily dengue case counts at the address level, spanning 8 years (2015–2022), to evaluate the importance of modeling human-mosquito interactions at an hourly resolution in elucidating sub-neighborhood dengue occurrence in the municipality of Rio de Janeiro. The findings of this urban study demonstrate that integrating knowledge of *Aedes* biting behavior with human movement patterns can significantly improve inferences on urban dengue occurrence. The inclusion of spatial eigenvectors and vulnerability indicators such as healthcare access, urban centrality measures, and estimates for immunity as predictors, allowed a further fine-tuning of the spatial model. The proposed concept enabled the explanation of 77% of the deviance in sub-neighborhood DENV infections. The transfer of these results to optimize vector control in urban settings bears significant epidemiological implications, presumably leading to lower infection rates of *Aedes*-borne diseases in the future. It highlights how increasingly collected human movement patterns can be utilized to locate zones of potential DENV transmission, identified not only by mosquito abundance but also connectivity to high incidence areas considering *Aedes* peak biting hours. These findings hold particular significance given the ongoing projection of global dengue incidence and urban sprawl.

## Introduction

The increasing amount of worldwide collected human movement data has a large potential to address current public health challenges^1–3^. Human mobility patterns, derived from a variety of data sources, such as mobile phone networks or social media platforms^4,5^, offer not only the ability to predict the spatial occurrence of infectious diseases^6–9^ but also to assess the effectiveness of control interventions^10,11^. This is of particular interest for many vector-borne diseases, for which labor-intensive vector control remains the most efficacious countermeasure^12–14^. Among them, the mosquito-borne disease dengue fever is the most important, with a 30-fold increase in incidence over the last 50 years, causing approximately 400 million infections each year^15^.

**Fig. 1:**
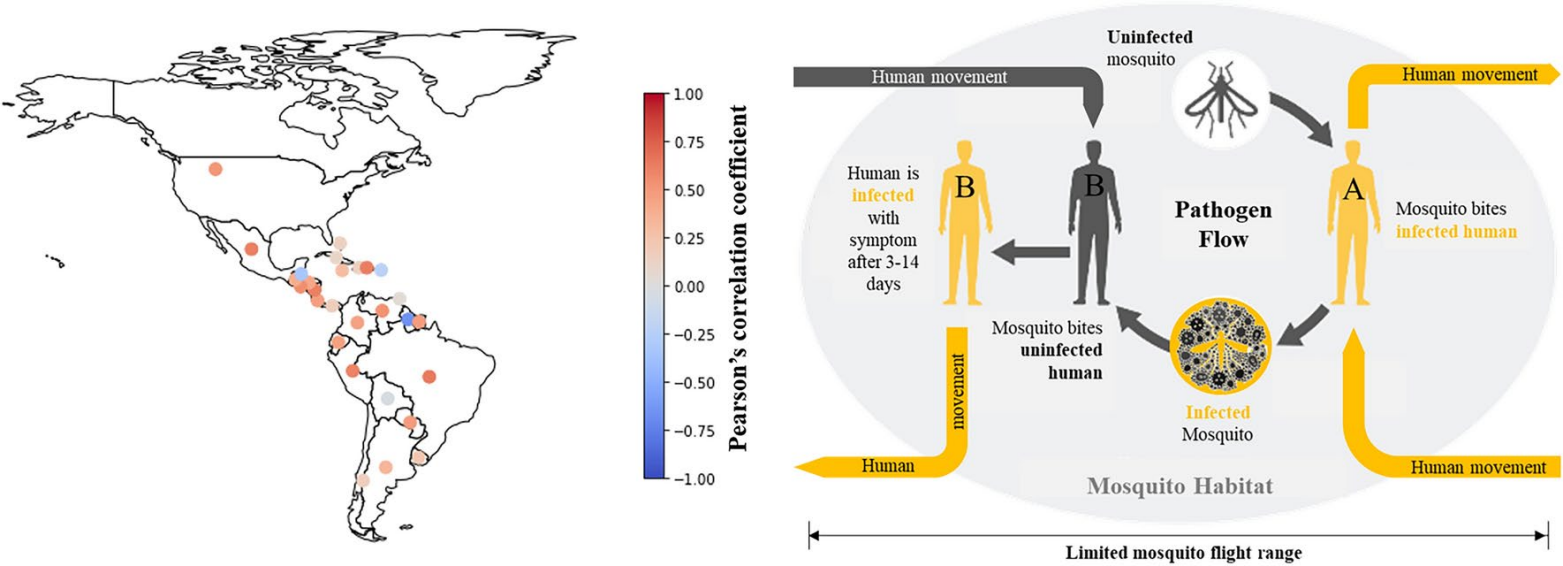
Pearson’s correlation coefficients between yearly dengue incidence^37^ and percentage share of population in urban areas^38^ for PAHO (Pan American Health Organization) countries between 1960 and 2021. This analysis explores the potential association between urban growth and the increase in global DENV occurrence, recognizing that correlation does not necessarily imply causation (left). Urban cycle of DENV transmission, highlighting the role of human movement and limited mosquito flight range for disease occurrence (right).

Urban growth, climate change, and international travel are known key drivers for this global incline in the dengue virus (DENV) occurrence (cf. Fig. 1)^16^. DENV transmission dynamics are highly determined by the interplay of mosquito abundance and connectivity, as defined by human movements^17,18^. A precise understanding of these risk factors, especially their spatial variation and interaction, is essential for an efficient allocation of vector control resources and the prevention of DENV outbreaks world wide^19–21^. Modeling human-mosquito interactions however can be a challenging task, especially at urban scale, where most DENV infections occur and precise knowledge about mosquito abundance as well as hourly human movement patterns is often missing^22^. This research work is part of the ’Lancet Commission on Dengue’ and aims to study the phenomenon of urban areas as global hotspots for dengue transmission and prevention.

Another challenging aspect of modeling human-mosquito interaction involves considering the ecological characteristics of the vector. DENV is a flavivirus transmitted primarily by female mosquitoes of the species *Aedes aegypti* and *Aedes albopictus*^23,24^. Both mosquito species tend to breed in small, artificial water containers often found in close proximity to human settlements^25–31^. Additionally, they exhibit a limited flight range, which is estimated to be below 1000 m^32–35^, and a diurnal biting behavior that mainly covers evening and morning twilights. Incorporating these ecological vector characteristics into the modeling of urban dengue outbreaks is imperative in the pursuit of alleviating the global burden of dengue fever^36^.

In this study, our primary objective is to assess the importance of ecological vector characteristics in explaining the spatial distribution of urban dengue infections. We propose that incorporating human-mosquito interactions on an hourly basis, while considering the diurnal biting behavior of mosquitoes and daytime commuting patterns of humans at a sub-neighborhood scale, may significantly impact our understanding of inner-urban dengue dynamics. To achieve this, we analyze the sensitivity of inferences related to various assumptions about hourly human-mosquito interactions. We develop two distinct modeling scenarios: one that neglects existing knowledge about *Aedes* mosquito twilight activity, and another that incorporates this knowledge through feature engineering, allowing for a more comprehensive analysis of the intricate dynamics of urban dengue infections. A low sensitivity to these inferences would suggest that ecological vector characteristics play a minor role in urban dengue outbreaks. Conversely, a high sensitivity would underscore the need to carefully account for diurnal biting behavior and daytime human movements when modeling DENV infections, especially at a fine-grained urban scale. In order to carry out this investigation we integrate data from previous research on high-resolution urban mosquito mapping^39^ and inner-urban human mobility patterns^40^, thereby creating hourly transmission risk maps. Our study focuses on the municipality of Rio de Janeiro, Brazil, an urban area endemic for *Aedes* mosquitoes and experiencing numerous dengue cases annually^41^. The findings from this research could significantly enhance our understanding of urban dengue transmission dynamics and potentially contribute to the development of more effective control strategies for this disease. More specifically, our investigation focuses on evaluating the impact of two key factors on model enhancement: (i) the feature engineering of hourly human-mosquito biting risk and (ii) the incorporation of spatial eigenvector mapping and vulnerability indicators.

## Materials and methods

**Fig. 2:**
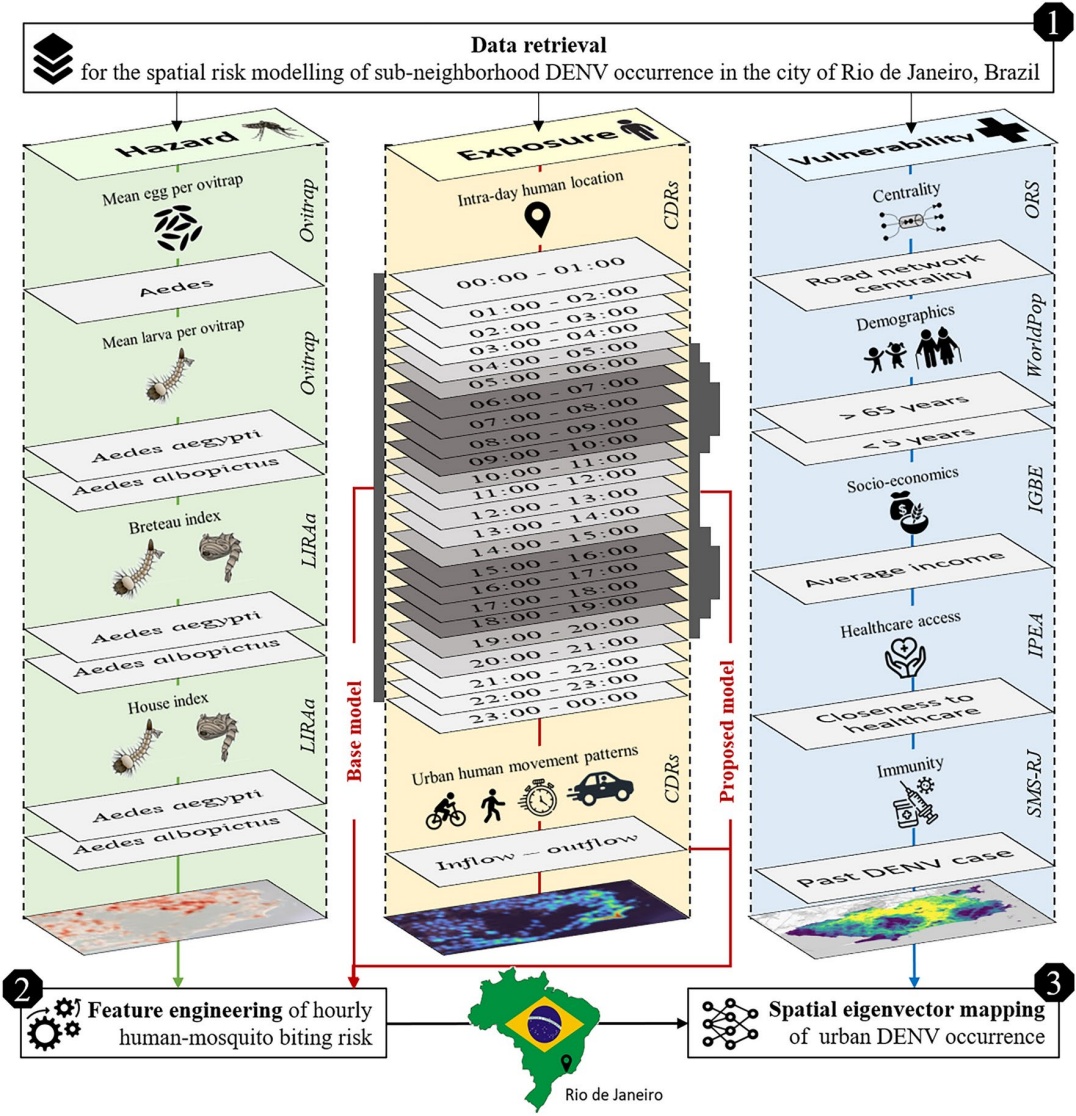
Workflow for the sub-neighborhood spatial eigenvector mapping of urban DENV occurrence applying entomological surveillance (left) and call detail records (middle) to model daytime human-mosquito biting risk for the municipality of Rio de Janeiro in Brazil on an hourly basis. Voronoi tessellations based on mobile phone antenna locations were employed as the spatial unit for analysis. In the feature engineering process, the base model assumed a constant human-mosquito interaction throughout the day, while the proposed model accounted for the fluctuating exposure of humans to mosquito bites, considering the twilight biting activity of *Aedes* mosquitoes and the hourly commuting patterns of humans. Note that this workflow identifies associations at an aggregate level and should be interpreted with caution to avoid ecological fallacies, as it does not imply causation at the individual level. (CDRs: Call detail records; ORS: OpenRouteService; IGBE: Brazilian Institute of Geography and Statistics; IPEA: Institute of Applied Economic Research; SMS-RJ: Municipal Health Ministry of Rio de Janeiro).

Here, we propose a novel risk modeling framework integrating ecological characteristics of *A. aegypti* and *A. albopictus* with data-driven insights on inner-urban human movement flows. This framework consists of three main parts (cf. Fig. 2): (i) the retrieval of DENV-related proxies capturing the three risk components of hazard, exposure, and vulnerability, (ii) the daytime feature engineering of human-mosquito biting risk integrating human movement data with knowledge of *Aedes* biting behavior, and (iii) the spatial eigenvector mapping^42^ of urban DENV occurrence using vulnerability indicators.

### Data

**Fig. 3:**
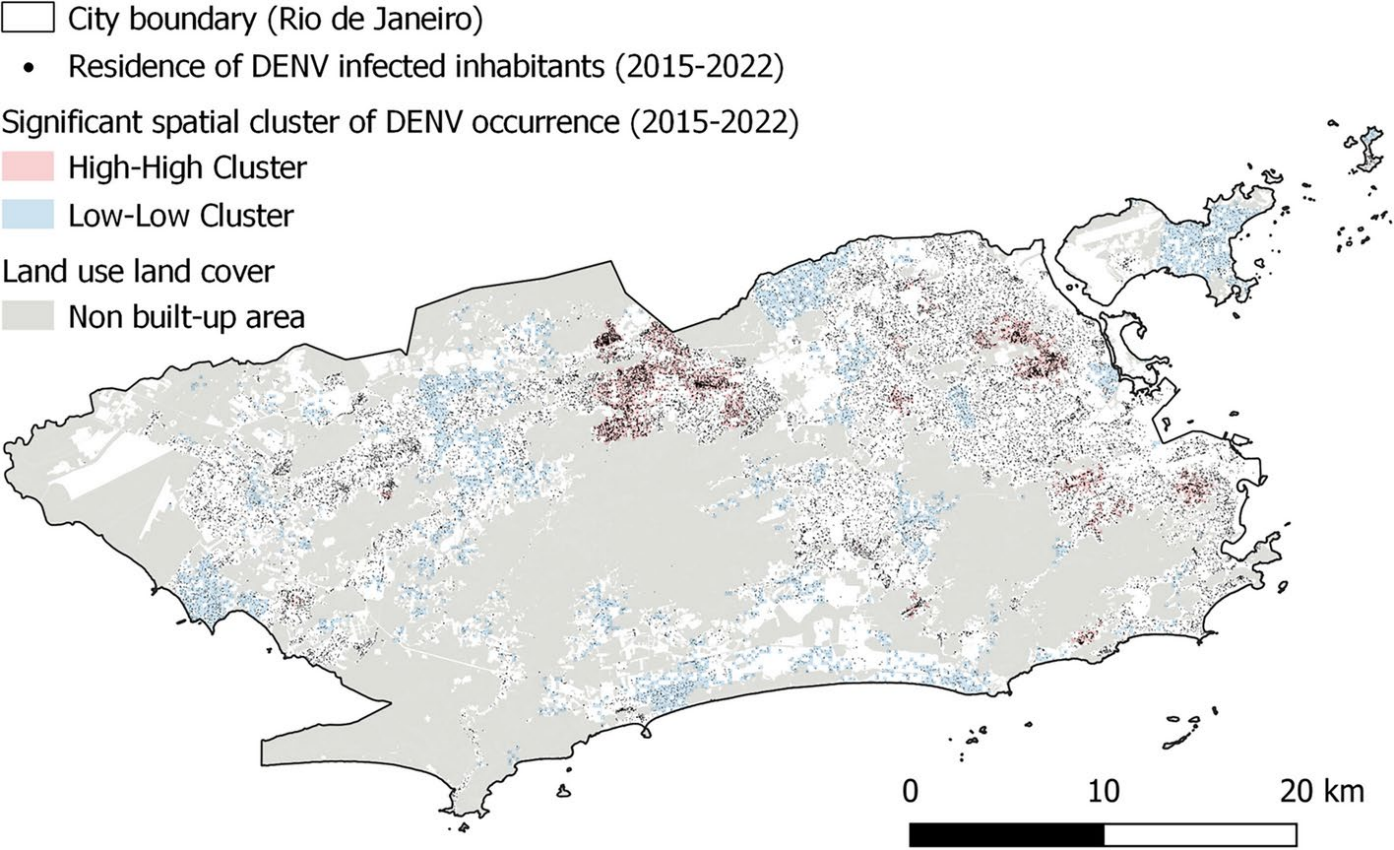
A 200 m grid displaying statistically significant hotspots, cold spots, and spatial outliers derived from daily DENV health records collected for the municipality of Rio de Janeiro between January 2015 and December 2022. Spatial autocorrelation and the identification of clusters with similar or dissimilar values were assessed using the Anselin Local Moran’s I statistic. In this context, ‘High–High’ clusters represent areas with high DENV occurrence surrounded by neighboring areas with similarly high occurrence, and ‘Low–Low’ clusters indicate areas with low occurrence surrounded by low-occurrence neighbors. Areas colored white indicate the absence of significant spatial autocorrelation in dengue occurrence. (created using ArcGIS).

All employed datasets, including their sources, spatial resolutions, and pre-processing procedures, are listed in Table 2. In order to evaluate our approach, we acquired daily counts of DENV cases from 2015 to 2022 with geographical coordinates corresponding to residential addresses (cf. Fig. 3). In adherence to ethical considerations and following approval by the Research Ethics Committee (CEP), this dataset underwent anonymization and was made accessible upon formal request by the municipal health ministry of Rio de Janeiro.

#### Hazard

The hazard risk components in this study were modeled using a variety of entomological surveillance data sources, focusing on different stages of the mosquito lifecycle. These data included mosquito egg and larva counts (cf. Fig. 7) as well as indices representing larva and pupa infestation (cf. Fig. 8). To model the abundance of immature *A. aegypti* and *A. albopictus* in urban areas, we used egg and larval counts from the year 2019, collected from 2698 ovitraps distributed across the study area. However, since ovitrap counts have limited spatial validity due to heterogeneous urban landscapes and the restricted flight range of *Aedes* mosquitoes, previous studies enhanced this dataset by incorporating high-resolution urban suitability indicators^22,39,43^. This approach allowed for a continuous approximation of seasonal urban mosquito suitability, considering a limited flight range of 200 m. For modeling larvae and pupae infestation, data from the Larval Infestation Rapid Assay (LIRA) were applied. These datasets covering the years 2015–2022 included seasonal Breteau and House indices, which were collected for 256 homogeneous street blocks defined by entomologists during survey design. The House index (HI) gauged the number of infested houses relative to the total visited buildings during LIRA, while the Breteau index (BI) represented the number of positive containers per 100 houses inspected. All entomological surveillance datasets used in this research were obtained from the municipal health department of the municipality of Rio de Janeiro upon request, exclusively for the purpose of this study.

#### Exposure

**Fig. 4:**
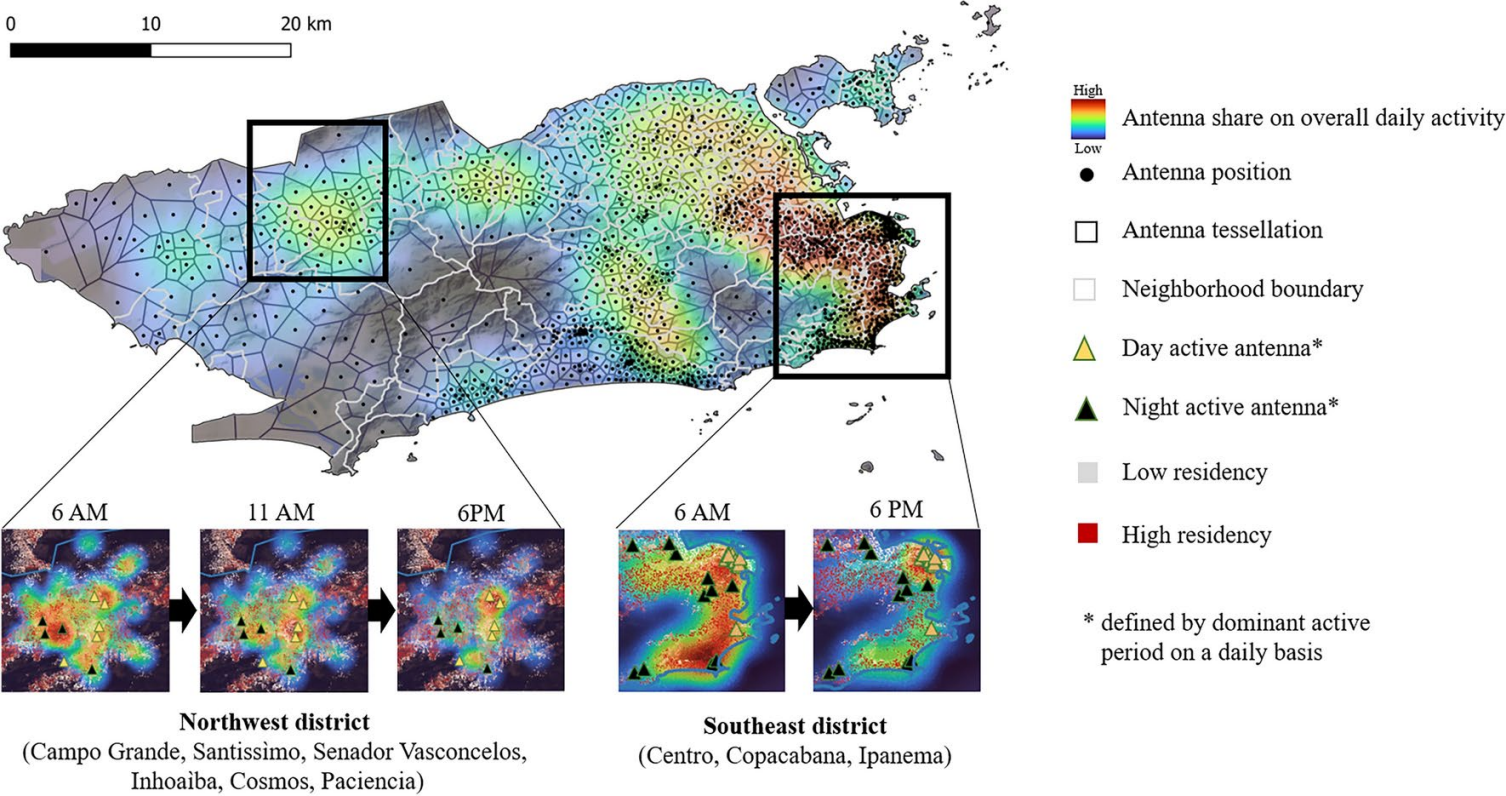
Daytime human population density in the municipality of Rio de Janeiro, estimated by using mobile phone data. Hourly changes in antenna activity behave differently in various zones of the case study region, as shown for two selected subregions. While the dominant mobility motif in the northwest district involved movement between three locations, the southeast district exhibited a dominant mobility motif characterized by movement between two locations. (created using QGIS).

To model the exposure components, we obtained hourly origin-destination (OD) matrices and corresponding population density maps using anonymized call detail records provided by a large Brazilian telecommunications company. This raw dataset comprised individual antenna connections from approximately three million individual users, representing an estimated 45 percent of the population of the municipality of Rio de Janeiro. The raw data had a temporal resolution of 5 min, capturing user connections to 1359 antennas distributed across 163 neighborhoods. In this study, the collective OD matrices for Voronoi tessellations, delineated by the locations of antennas, were generated based on the temporal sequences of individual antenna connections spanning from July 2021 to July 2022, encompassing a complete annual cycle of human mobility patterns. A more extensive description of the applied methods was given by a previous study^40^. Figure 4 illustrates the fluctuations in human population density throughout the day due to commuting dynamics within the municipality of Rio de Janeiro, where day and night active antennas are defined by their dominant active periods on a daily basis.

#### Vulnerability

We hypothesized that the likelihood of an infected individual appearing in official health registries is influenced not only by the human-mosquito biting risk but also by other factors, which were defined as vulnerability indicators and considered to refine the precision of estimating the spatial distribution of dengue cases, especially within an urban setting. The utilized indicators can be classified into five subgroups: centrality, accessibility, socio-economics, demographics, and level of immunity. Centrality indicators were derived from OpenStreetMap (OSM) using the OpenRouteService API^44^. Mean road centrality by average travel time was calculated on a 200m grid using cars as a transportation medium (cf. Fig. 9). These measures were hypothesized to estimate human closeness and interaction, as tested in a previous study^45^. Accessibility to job opportunities and travel time to the closest healthcare facilities using active transportation as well as public schools were retrieved from the Institute for Applied Economic Research (IPEA) on a hexagonal grid of 0.11 km^2^ (cf. Fig. 10)^46^. The same data source was applied to download a cumulative opportunity measure of the whole population, indicating the number of opportunities that can be reached within 60 minutes of travel time, and the socio-economic indicator of average household income per capita. We hypothesized that all these accessibility and socio-economic indicators influence the appearance of dengue infections in the official health database. For instance, we assumed that inhabitants of shanty towns (favelas) in Rio de Janeiro with lower average income and lower accessibility measure are less likely to visit a doctor with dengue symptoms compared to people of higher social class^47,48^.

Information about the most vulnerable age groups for DENV infections was included by using population estimates for children below five years and elderly individuals above 60 years from the Humanitarian Data Exchange^49^. These indicators were included to estimate the severity of symptoms^50^ and, thus also the likelihood of visiting a doctor and being registered in an official health database (cf. Fig. 11). Additionally, we retrieved the locations of past DENV infections, including all four DENV serotypes, hypothesizing that past dengue epidemics serve as a reliable indicator for modeling immunity levels at the population level^51^. However, this immunological vulnerability effect would likely be complex as it is dependent on the sequence of DENV serotypes causing DENV cases over time as well as the time intervals between them^52^. All mentioned vulnerability components were combined with the daytime models for human-mosquito biting risk to facilitate spatial eigenvector mapping of urban DENV occurrence, which is described in the following “Methods” section.

### Methods

#### Feature engineering of hourly human-mosquito biting risk

In this study, we propose a novel method to model the spatial distribution of human-mosquito biting risk in urban areas by incorporating ecological characteristics of mosquitoes, specifically focusing on the diurnal biting behavior of *A. aegypti* and *A. albopictus*. Our method involved integrating local estimates of mosquito abundance $M_i$ with knowledge about hourly human distribution over the considered area to derive an aggregate measure of mosquito biting risk $B_i$ for residents of cell $C_i$:

1 $$\begin{aligned} B_i = \left[ \sum _{h=1}^{24} \omega (h) \left( \sum _{j=1}^N \chi _{i,j}(h) M_j \right) \right] \end{aligned}$$

Equation 1 aims to more accurately reflect mosquito bite risk than estimates solely based on local mosquito abundance by incorporating two key principles. First, due to human movement, individual hosts are exposed to different mosquito populations throughout the day. To capture this for each hour of the day *h*, we calculated a weighted sum approximating the contribution of mosquito populations $M_j$ from all cells $C_j$ to the biting risk of people resident in cell $C_i$. This sum reflects the extent to which the hourly mosquito biting risk originating from the mosquito population $M_j$ in cell $C_j$ affects individuals residing in cell $C_i$. To this end, we estimated $\chi _{i,j}(h)$, representing the fraction of people present in antenna tessellation cell $C_j$ during hour *h*, relative to the total number of residents in antenna tessellation cell $C_i$. The calculation of $\chi _{i,j}(h)$ utilized hourly OD matrices, indicating collective human mobility from cell $C_i$ to cell $C_j$. Secondly, considering the daytime variation in mosquito biting behavior, we introduced the hourly weighting function, denoted as *w*(*h*) in our model (cf. Eq. 2). It is well-documented that *A. aegypti* and *A. albopictus* biting behavior occurs exclusively during daylight hours, with heightened activity observed during twilight^53–55^. As such, we assumed a decrease in mosquito biting activity during midday hours. However, we posited that this behavior might persist in shaded regions characterized by elevated humidity and other environmental factors favoring mosquito activity^56–58^. Notably, mosquito biting activity during the night was excluded from our proposed model.

2 $$\begin{aligned} \begin{aligned} \quad \omega (h)= \left\{ \begin{array}{ll} 3, & \text {if}\ h \in \{6,7,8,9,15,16,17,18\} \\ 2, & \text {if}\ h \in \{5,10,14,19\} \\ 1, & \text {if}\ h \in \{4,11,12,13,20\} \\ 0, & \text {otherwise} \\ \end{array} \right. \end{aligned} \end{aligned}$$

The proposed feature engineering underwent evaluation employing a quasi-Poisson generalized linear model (QP-GLM), wherein the target variable $D_i$ was defined by overdispersed official dengue case counts aggregated on 1359 antenna tessellations between the years 2015 and 2022 (cf. Eq. 3). For evaluation, we calculated Cohen’s pseudo-$R^{2}$ (cf. Eq. 4). The explained deviance for this regression model was compared to the pseudo-$R^{2}$ of a base model that did not consider assumptions related to diurnal *Aedes* mosquito biting behavior and hourly human movement (cf. Fig. 2). In contrast to the proposed model, the base model was implemented utilizing identity OD matrices for $\chi _{i,j}(h)$.

3 $$\begin{aligned} & \begin{array}{c} D_i \sim \text {quasi-Poisson}(\hat{\mu _i},\hat{\theta })\\ \mathbb {E}(D_i)=\hat{\mu _i}\\ \textrm{Var}(D_i)=\hat{\mu _i} *\hat{\theta }, \text { with } \hat{\theta } \ne 1\\ \log (\hat{\mu _i}) = \log (H_i)+\hat{\beta _0} + \hat{\beta _1} *B_i \end{array} \end{aligned}$$

4 $$\begin{aligned} & \begin{array}{c} Cohen's\ pseudo\ R^2 = 1 - \frac{model\ deviance}{null\ model\ deviance}\\ \end{array} \end{aligned}$$

#### Spatial eigenvector mapping incorporating selected vulnerability indicators

After evaluating the feature engineering of hourly human-mosquito biting risk, we expanded our QP-GLM in two aspects: (i) by incorporating vulnerability indicators to model the likelihood of an infected individual being registered in official health registries, geolocated by residency, and (ii) by integrating spatial eigenvectors to address spatial autocorrelation of residuals. To mitigate multicollinearity among covariates, we selected vulnerability indicators with low intercorrelation ($\le$ 0.7). These two model extensions led to a more comprehensive model for sub-neighborhood DENV occurrence, considering daytime human-mosquito biting risk, as explored in our initial research question.

Our second research objective focuses on the enhancement of spatial estimates of sub-neighborhood DENV occurrence by incorporating spatial eigenvectors and selected vulnerability indicators. By addressing this question, we aim to assess the extent to which these additional variables improve the predictive capability and understanding of DENV transmission dynamics within the urban environment. Here, vulnerability features were defined as variables that influence the appearance and collection process of DENV infections at the urban scale, but not the human-mosquito biting risk itself. This strategic inclusion allows us to dissect the nuanced factors contributing to DENV occurrence, beyond solely focusing on the dynamics of human-mosquito interactions. In this study, these factors included the location of vulnerable age groups, accessibility to health care facilities, road network centrality, the socio-economic factor of average income, and estimates on immunity levels derived from past DENV infections. In contrast to the first model defined in Eq. 3, the year 2022 was selected as the reference year for predictions, coinciding with the occurrence of the last major DENV outbreak in the municipality of Rio de Janeiro (cf. Fig. 12). Consequently, immunity levels were estimated based on the spatial distribution of past infections recorded between 2015 and 2021.

**Fig. 5:**
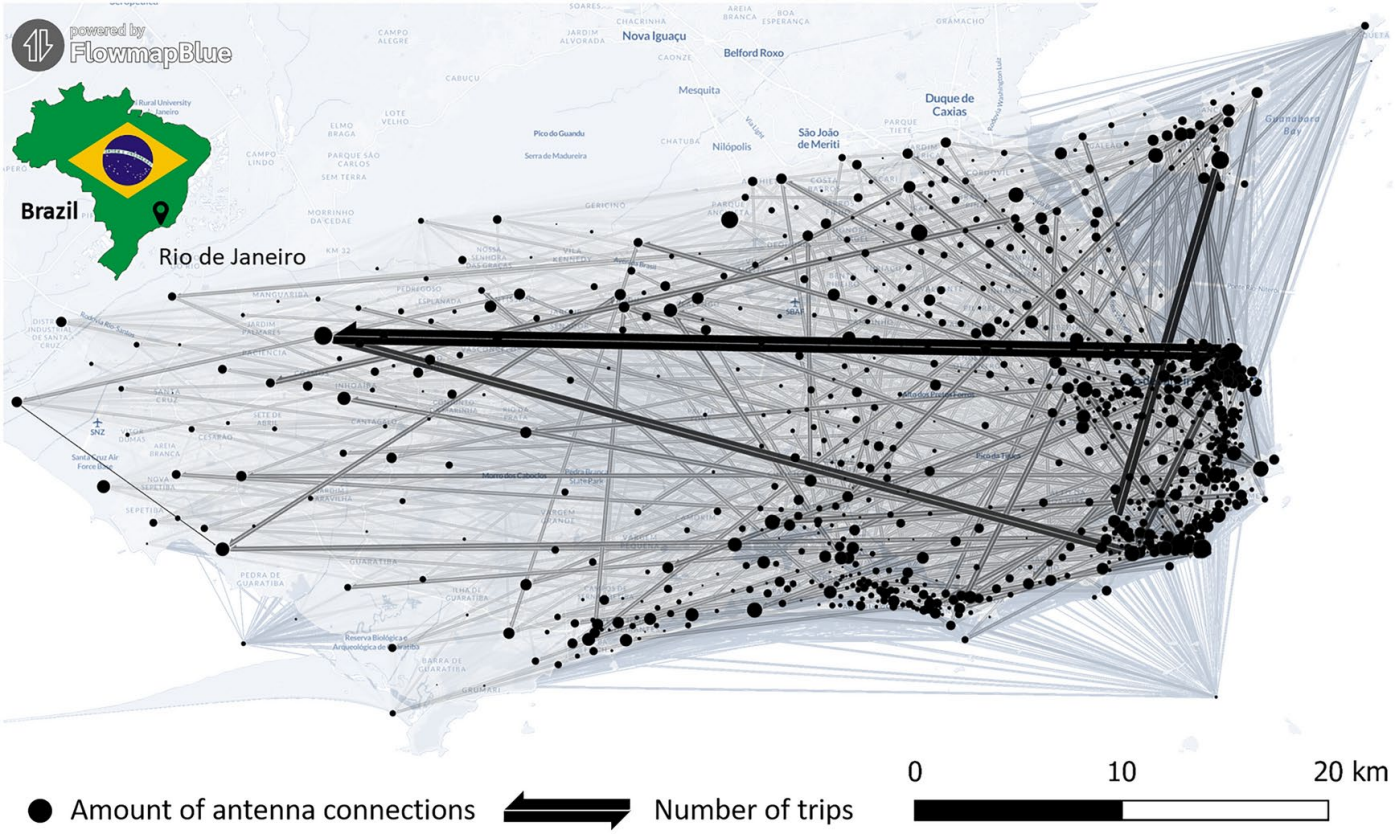
Human movement patterns used for spatial eigenvector mapping of DENV occurrence in the municipality of Rio de Janeiro. Spatial weights were estimated applying mobile phone records from July 2021 to July 2022. Thick dark black edges represent high human connectivity between antenna locations, whereas thin and bright black stripes indicate a lower amount of human movements. (created using FlowmapBlue).

The applied spatial eigenvector mapping, originally proposed by Griffith et al.^42^, involved the incorporation of additional covariates to absorb spatial autocorrelation. This ensures unbiased estimators for other predictors. These covariates, derived from the eigenfunction decomposition of the spatial weight matrix *W*, are called spatial eigenvectors. They represent orthogonal components that effectively separate and capture information on spatial autocorrelation, similar to principal component analysis. In our study, we employed daily aggregated OD matrices from July 2021 to July 2022 to illustrate human connectivity between antenna tessellations, serving as a spatial weight matrix (cf. Fig. 5). This led to the generation of 1359 spatial eigenvectors, out of which the ME function from the ’spatialreg’ R package facilitated the identification of a specific subset applying brute-force search^59,60^ under consideration of an alpha threshold of 0.05 to mitigate residual autocorrelation. This selected subset of eigenvectors was integrated as additional covariates into the QP-GLM (cf. Eq. 3).

## Results

### Evaluating the feature engineering of hourly human-mosquito biting risk

The results in Table 1 demonstrate how considering mosquito biting hours and human movement corridors can enhance the accuracy of spatial estimates for urban DENV occurrence. The proposed feature engineering method outperforms the baseline model, which does not consider the daylight activity of *Aedes* mosquitoes, and demonstrates a 13.5% increase in the explained deviance within the response of the QP-GLM. Both models yielded positive and significant estimates for their hazard and exposure combined covariate of human-mosquito biting risk $B_i$. The computed global Moran’s I value for the residuals was 0.59.

**Table 1:** Coefficients, standard errors, and p-values for QP-GLMs applying two different model scenarios for human-mosquito interaction, where the base model did not consider any temporal variation in human-mosquito biting risk and the proposed model incorporated hourly adapting mosquito activity and human population densities. Regression coefficients and standard errors are reported at the link scale. The limited explained deviance in both models hints at the presence of missing latent covariates.

QP-GLM	Intercept		Human-mosquito biting risk ($B_i$)			Cohen’s explained deviance
	$\hat{\beta _0}$	$\hat{\sigma }_{\hat{\beta _0}}$	$\hat{\beta _1}$	$\hat{\sigma }_{\hat{\beta _1}}$	$Pr(>|z|)$	pseudo-$R^{2}$
Base model (BM)	− 0.7914	0.2856	2.3228	0.4591	$4.78e^{-7}$	0.0395
Proposed model (PM)	0.4273	0.0532	4.2866	0.2230	$< 2e^{-16}$	0.1750

Considering the aforementioned results, it implies that integrating knowledge of *Aedes* biting behavior with human movement patterns can also facilitate the inference of probable transmission sites for reported dengue cases. If this holds true, increased mosquito control interventions in these locations would have the potential to combat *Aedes*-borne diseases more effectively.

**Fig. 6:**
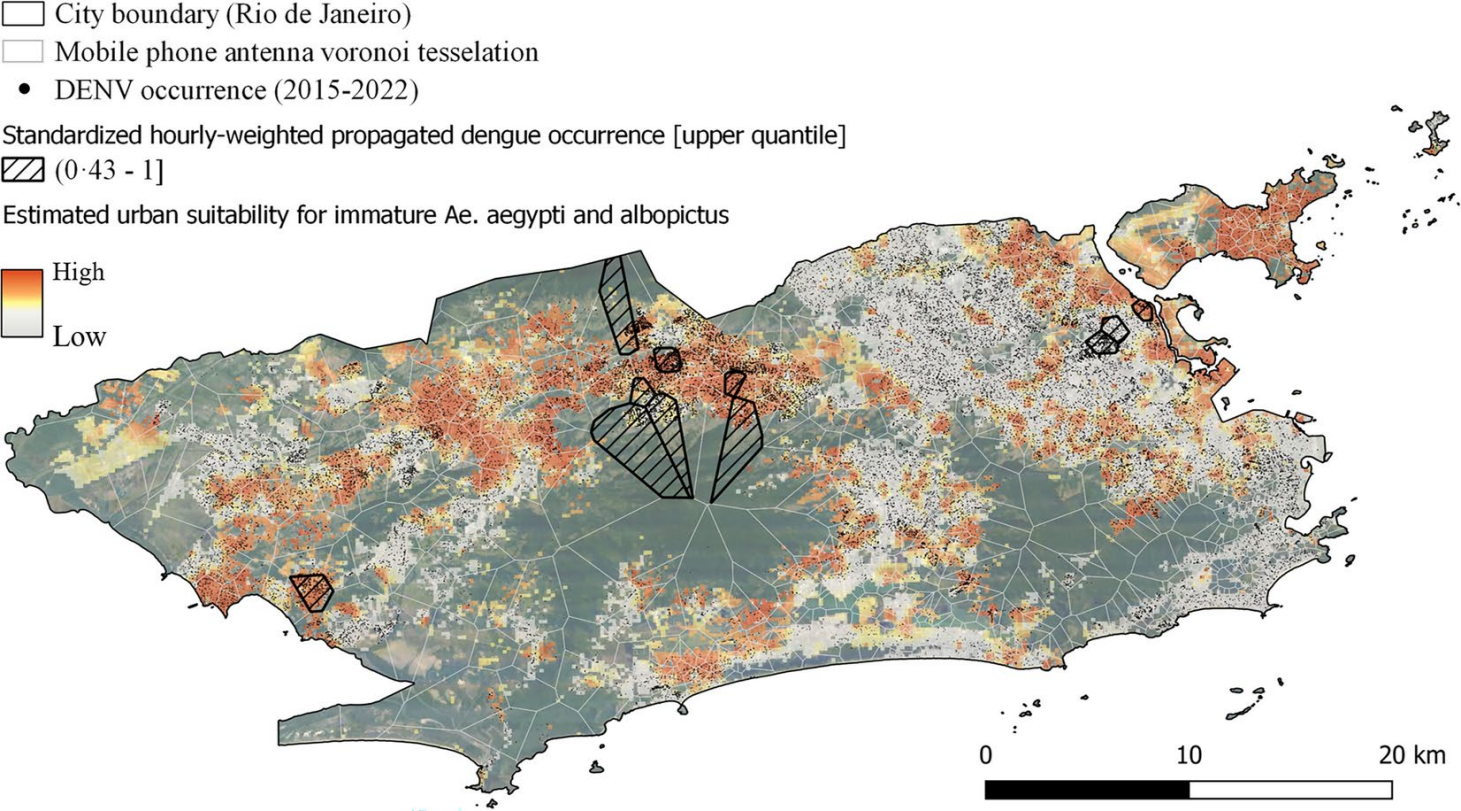
Novel vector control planning map considering daytime mosquito activity and human movement flows for the municipality of Rio de Janeiro. The figure illustrates the discrepancy between DENV occurrence and estimated mosquito abundance at an urban scale. Areas of dark red color represent target effectiveness zones measured by entomological surveillance. The black-striped Voronoi tesselations highlight potential danger areas for transmission that might be underestimated when relying solely on entomological surveillance or reported dengue cases. The identification of these zones relied on hourly-weighted propagated dengue occurrence $HP-DENV_{i}$, weighted by biting activity, to reflect the locations of infected persons during the days denoted as $HP-DENV_{i} = \sum _{h=1}^{24} w(h) \cdot \left( DENV \cdot \prod _{j=1}^{h} OD_j \right)$. Within the black-striped Voronoi tessellations, sub-regions with high mosquito suitability are particularly relevant to guide interventions. (created using QGIS).

Figure 6 presents, as a highlight of this work, the practical implications of these research findings for the municipality of Rio de Janeiro. A novel mapping approach for vector control intervention was developed, incorporating (i) the spatial distribution of mosquitoes, as indicated by temporally aggregated entomological surveillance data, (ii) the spatial dispersal of dengue occurrence, and (iii) the most likely transmission locations for reported dengue cases, taking into account daytime *Aedes* biting behavior. This target effectiveness map marks regions that were potentially underestimated for vector control planning using entomological datasets only, while at the same time emphasizing the enduring importance of areas with high mosquito abundance.

### The role of vulnerability indicators and spatial eigenvector mapping in model enhancement

**Fig. 13:**
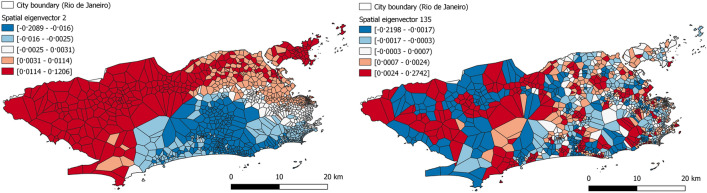
Exemplary spatial eigenvectors derived from the model’s spatial weight matrix illustrating distinct patterns: Spatial eigenvector 2 shows gradual spatial gradients, and spatial eigenvector 135 depicts localized clustering. These eigenvectors unveil varying spatial structures within the study area, providing valuable insights into the underlying spatial relationships influencing the observed phenomena. (created using QGIS).

We hypothesized that incorporating vulnerability indicators and spatial eigenvectors would further enhance the proposed QP-GLM (cf. Equation 3), which considers *Aedes*-human interactions for predicting the spatial occurrence of dengue in the municipality of Rio de Janeiro. The Cohen’s pseudo-R2 of the more extensive QP-GLM considering hourly human-mosquito biting risk was determined to be 0.77, indicating that the extended model was capable of explaining up to 77 percent of the deviance in dengue occurrence on the sub-neighborhood level for the municipality of Rio de Janeiro in the year 2022. The computed global Moran’s I value for the residuals was 0.07, indicating low spatial autocorrelation. A QP-GLM with human-mosquito biting risk and vulnerability indicators but without spatial eigenvectors was not considered, as it yielded a higher overdispersion value of 26$.$85 and a higher global Moran’s I of 0.2, despite having a Cohen’s pseudo-R2 of 0.83. This underscores the importance of vulnerability indicators and spatial eigenvector mapping in improving spatial predictions of sub-neighborhood dengue occurrences, which are georeferenced based on residency. Additional result on the applied vulnerability indicators and spatial eigenvectors are listed in the Appendix 2 (Table 3, Fig. 13).

## Discussion

In this study, we analyzed the impact of modeling urban DENV occurrence under consideration of daytime mosquito activity and human movement patterns. The analysis has shown how urban areas exhibit spatial heterogeneity in numerous factors relevant to infectious disease transmission. The findings contribute to the understanding of infectious disease dynamics at a sub-neighborhood scale by highlighting the important role played by daytime mosquito activity and human movement flows in linking observed patterns of DENV incidence to inferred patterns of disease transmission. The inferred degree of spatial variation in urban DENV occurrence was sensitive to assumptions about daytime mosquito activity. Spatial discrepancy existed between the dominant location of mosquitoes, the spatial patterns of human-mosquito interaction points, and disease occurrence collected by residency. Taking these findings into account, one can conclude that methodologies that presume consistent human exposure to mosquito bites throughout the day potentially yield exaggerated and biologically inadequate interpretations regarding the patterns of disease transmission.

### Hazard

We modeled hazard risk components associated with *Aedes* mosquitoes utilizing various entomological surveillance data. However, several limitations affect the reliability of our findings. First, the resolution of the LIRA data impedes the ability to accurately capture the high spatial variability of *Aedes* that may occur in heterogeneous urban landscapes such as Rio de Janeiro, owing to the limited flight range of mosquitoes. Second, while we incorporated entomological data on eggs, larvae, and pupae, the absence of data on adult mosquito populations is a notable limitation of this study. Adult populations are crucial for understanding transmission dynamics; thus, future research should address this gap by implementing monitoring strategies for adult populations, potentially using smart traps to yield more comprehensive data. Additionally, the ovitrap surveillance from 2019 does not fully align with the analysis timeframe of 2015 to 2022. This temporal discrepancy primarily stems from restricted access to the ovitrap data, which required ethical approval for this research. This limitation highlights the need for a culture of open data sharing to facilitate further research in the field of eco-epidemiology, ultimately enhancing our understanding of dengue transmission dynamics and potentially improving control interventions in the long term. In summary, addressing these uncertainties through improved methodologies and robust data collection will be essential for accurately modeling the hazards associated with dengue transmission.

### Exposure

While mobile phone data significantly improved predictive accuracy, potential biases may have been introduced by excluding individuals without mobile phones or those using alternative service providers. Another limitation is related to the spatial resolution of the analysis, as mobile phone records could only be georeferenced by antenna tessellation, which may have affected spatial precision. Future studies could address this by validating the findings using higher-resolution mobility data, such as GPS trajectories,enabling modeling at an individual scale rather than aggregating risk into areal units, thus mitigating issues related to the Modifiable Areal Unit Problem (MAUP). Additional preprocessing challenges, such as accounting for offline movements and antenna congestion, further complicated the accurate extraction of human movement patterns. While upper and lower boundaries for inter-event times were implemented to mitigate data bias^40^, future research could explore the impact of these parameter choices on the retrieval of human movements from mobile phone data. Incorporating cross-boundary human movements to out-of-city regions, which were not included in this study, could additionally contribute to refining exposure estimates and enhancing overall predictive accuracy.

Our study models spatial dengue occurrence from January 2015 to December 2022; however, the exposure modeling component utilizes movement data only from July 2021 to July 2022. This discrepancy in timeframes may introduce biases, as the movement data do not fully represent the entire study period. Moreover, the 2021-2022 period overlaps with the COVID-19 pandemic, during which movement restrictions, quarantines, and behavioral changes likely influenced mobility patterns. These pandemic-related biases could impact the accuracy of our inferred human-vector interactions and associated dengue risk patterns. Future studies could improve alignment with the study period and reduce uncertainty in exposure estimates by incorporating a longer, non-pandemic timeframe for exposure modeling.

Given the restricted access to mobile phone data, Knoblauch et al.^40^ evaluated the use of geotagged tweets as a more openly available data source for modeling human movement patterns. The corresponding findings revealed the need for caution when using Twitter/X data for short-term urban mobility modeling, as it is vulnerable to policy changes and fluctuations in the availability of publicly accessible geotagged tweets. However, the 27-month validation study demonstrated that combining multiple mobility metrics, analyzing both dynamic and static mobility changes, and employing robust preprocessing techniques - such as rolling window downsampling - can improve the inference capabilities of Twitter/X data. Nevertheless, despite the application of these advanced methods, Twitter/X data may not always perform as well as mobile phone records in capturing human movement patterns, as validated during the COVID-19 pandemic using the stringency index, which measures the strictness of government-imposed mobility restrictions^40^. Future research could build on this validation study by exploring the potential of additional openly available data sources for retrieving human movement patterns. These may include data from public transit systems, ride-sharing apps, delivery services, household surveys, wearables, smart city sensors, and volunteered geographic information from platforms like Strava and Waze. Such data sources may offer valuable alternatives to mobile phone data and could provide critical insights into movement dynamics, essential for accurately modeling the spread of infectious diseases. Higher-order descriptions of movement, such as social network structure, have been shown to affect transmission dynamics in urban environments^18,61,62^. The consideration of the interplay among disease symptoms, infectiousness, and the mobility of individuals infected with DENV seems additionally promising in this context^63–65^. This complicates the assumption that the movement patterns of apparently healthy individuals can adequately represent the mobility patterns of those involved in transmission^66^.

### Vulnerability

Additional limitations of our analysis stem from uncertainties related to vulnerability indicators. Socioeconomic status data, aggregated at the census block level, may fail to capture intra-block heterogeneity, potentially introducing spatial biases when aligned with our analysis units based on antenna tessellation. Healthcare accessibility, modeled using the OSM road network and active transportation assumptions, likely oversimplifies access in real-world conditions. In practice, accurate accessibility assessments would benefit from a more comprehensive road network, as OSM may not capture all routes, and should also consider factors such as health insurance coverage and passive transportation options. Furthermore, the absence of data on vaccine trials or public health interventions targeting *Aedes*-borne diseases may introduce local immunity effects not accounted for in this study, as immunity here was modeled solely through DENV occurrence data. Future studies incorporating high-resolution socioeconomic metrics, refined healthcare accessibility measures, and records of intervention efforts could presumably reduce some uncertainties associated with vulnerability factors.

### Dengue occurrence

Our study leverages high-resolution dengue occurrence data at the point level, representing the precise latitude and longitude of each infected individual’s home address. This level of granularity enables integration with antenna tessellation cells, which we use as the spatial units of analysis (1359 cells in our study region), facilitating a finer-scale assessment of dengue transmission dynamics across the municipality of Rio de Janeiro. This approach marks a improvement over most studies, which are generally limited to the highest resolution available for openly accessible health records - typically aggregated at the neighborhood level in Brazil (e.g., 163 neighborhoods in our study region). However, the quality of this occurrence data is limited by several factors. First, underreporting may be more prevalent in areas controlled by milícias - paramilitary groups that govern nearly half of the city and impose alternative governance structures, including control over healthcare services^67^. In these regions, residents are often required to pay milícias a commission for essential services, including medical care. Such governance by milícias can lead to inconsistent or unverified case reporting, thereby introducing bias into official municipal health records. Additionally, misdiagnosis remains a concern, as symptom overlap with other mosquito-borne diseases, such as Zika and chikungunya, can lead to misclassification of dengue cases. This issue was further exacerbated during the COVID-19 pandemic, which coincided with our study period, when individuals presenting with fever and other overlapping symptoms may have been misdiagnosed or hesitant to seek medical care. Lastly, reporting delays resulting from bureaucratic processes within the public health system may affect the completeness and timeliness of the occurrence data.

### Modeling

Another key limitation of the results lies in the reliance on two relatively basic, temporally static statistical models for *Aedes*-human interactions: one assuming constant human exposure to mosquito bites throughout the day, and the other accounting for diurnal fluctuations. While these models offer valuable insights for comparing theoretical frameworks, they fall short of fully capturing the complexity of transmission dynamics. Future research could address this limitation by developing spatial process-based models that simulate transmission dynamics^68,69^. Though computationally demanding, such models would allow for the integration of mosquito behavior, ecological factors, and feedback mechanisms, including immunity dynamics and transmission cycles, to provide a more comprehensive understanding. In addition, incorporating the daytime variation in human host density across urban areas could enhance the models’ capacity to reflect changes in local vectorial capacity, as fluctuations in mosquito biting behavior and mosquito-to-host ratios could influence transmission risk. This approach would offer more refined insights into the efficacy of prevention and control strategies, presumably improving disease management in urban environments to a greater extent, as supported by the findings presented here. Besides that, future research could prioritize the development of spatiotemporal models that directly integrate high-resolution environmental suitability data and mobile phone data, rather than relying on spatiotemporal feature engineering. Adapting the proposed models to different mosquito species or regions will require accounting for variations in biting behavior influenced by factors such as day length. Incorporating seasonal shifts in sunrise and sunset times could further refine risk predictions, as could modeling partial nighttime activity of *Aedes* due to artificial lighting, particularly indoors. Smart traps equipped with optical or acoustic sensors provide a means to capture detailed, location-specific biting behavior of *Aedes*, offering more accurate data for modeling than the generalized literature values used in this study. Moreover, incorporating pathogen penetration rates for both host and vector populations into the models, may enhance the predictive accuracy of dengue occurrence models. However, widespread testing is often constrained by high costs, logistical complexities, and limited availability of advanced laboratory infrastructure.

## Conclusion

Overall, these findings underscore the critical importance of integrating vector ecology and human behavior into advanced disease modeling frameworks. A major challenge persists due to the lack of high-resolution data, which is essential for capturing real-world dynamics and enabling biologically sound interpretations of transmission patterns within eco-epidemiological models. While our analysis provides valuable insights, it is important to note that observed associations do not imply causation, given the inherent limitations of aggregate data and the potential for fallacies in interpretation. This emphasizes the need for caution in interpreting population-level patterns as causal relationships at the individual level. Although focused on dengue, the insights from this study may extend to other vector-borne diseases in which human movement patterns influence both pathogen dissemination and exposure to vectors. Future research could build on these findings by refining the proposed integration of human mobility data into vector control strategies. Shifting from a primary focus on vector abundance to guidance informed by vector-host interactions acknowledges that risk is shaped by the combined factors of hazard and exposure. Validating the effectiveness of these targeted vector control strategies through field studies across diverse geographical contexts will be essential for advancing their applicability beyond the specific case of Rio de Janeiro.

## Figures and Tables

**Figure 7:**
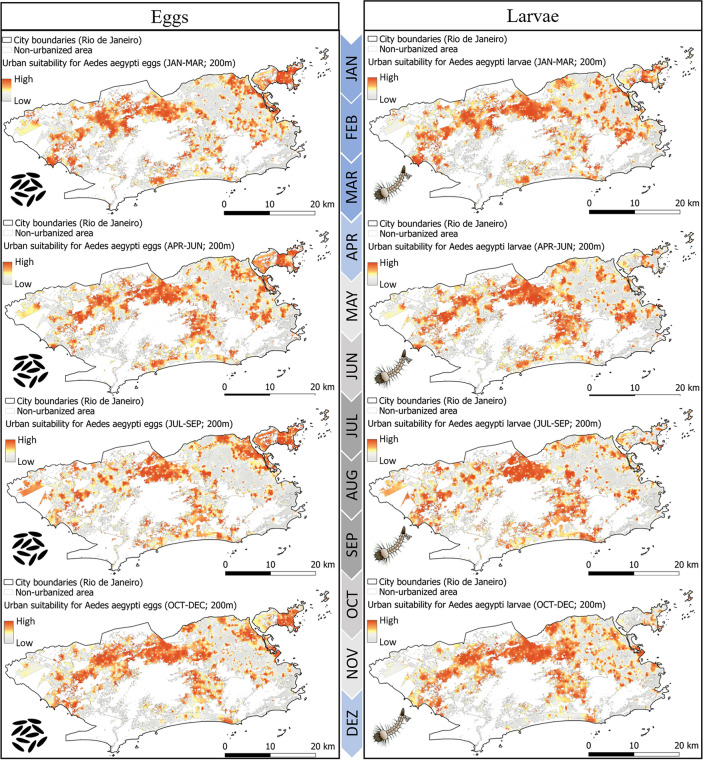
Seasonal urban suitability for *A. aegypti* eggs (left) and larvae (right) at a 200-meter resolution within the municipality of Rio de Janeiro for the year 2019, generated in a prior study^39^. The analysis employed a complementary approach integrating entomological surveillance data from ovitraps, ecological knowledge concerning limited mosquito flight range, and urban landscape indicators relevant to infer immature *A. aegypti* suitability. The blue timescale on the left indicates the wet and dry season. (created using QGIS).

**Fig. 8:**
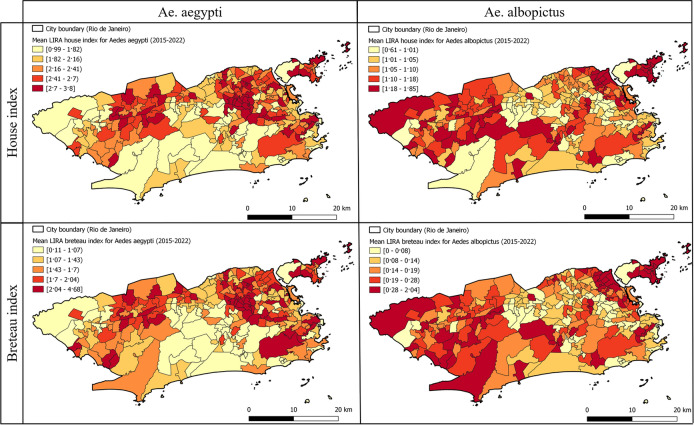
Entomological surveillance data collected via household survey called LIRA.MinisteriodaSaudeBrazil.2013 Maps display the house index (left) and breteau index (right) for *A. aegypti* (top) and *A. albopictus* (bottom) averaged over 48 seasonal LIRA surveys between 2015 and 2022. (created using QGIS).

**Fig. 9:**
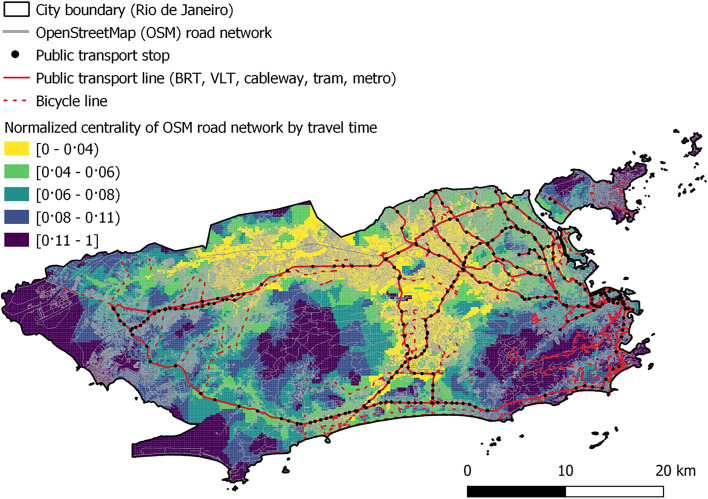
Normalized centrality of OSM road network measured by travel time in car for the municipality of Rio de Janeiro. (created using QGIS).

**Fig. 10:**
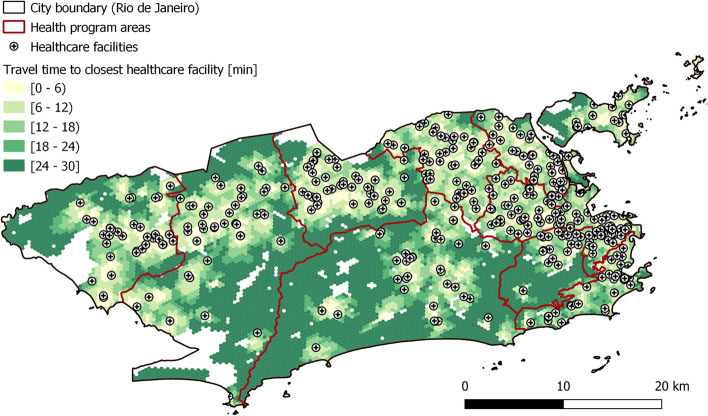
Healthcare accessibility measured by travel time using an equal modal split of car, public transport, bicycle and walk for the municipality of Rio de Janeiro. (created using QGIS).

**Fig. 11:**
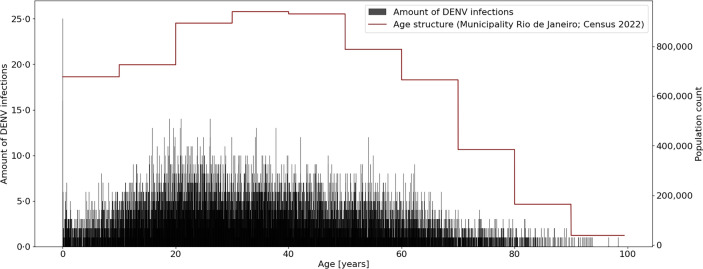
Comparison between the age distribution of DENV-infected individuals, determined by the time interval between birth and notification date in the official health system, and the overall age structure in Rio de Janeiro municipality as per the 2022 census. For the calculation of the average infection age, official health records from January 2015 to December 2022 were applied. The age structure of infected persons roughly aligns with the general demographic structure.

**Fig. 12:**
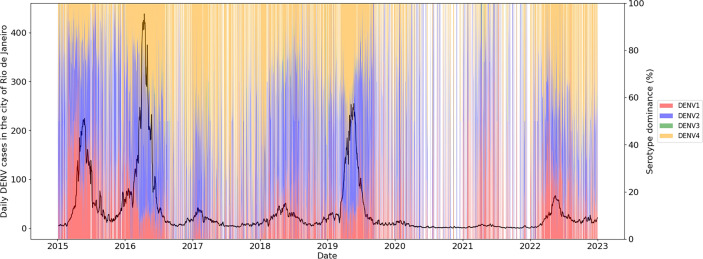
Daily fluctuations of DENV case counts and serotype dominance in the municipality of Rio de Janeiro from 2015 to 2022. Larger outbreaks in 2015, 2016, and 2019 co-occur with dominant serotype switches. Days indicated by white stripes indicate a lack of serotype tests in the official health database; many of these gaps also coincide with the COVID-19 pandemic.

## Data Availability

The materials and datasets generated and analyzed during the current study are available from the corresponding author upon reasonable request. Restrictions apply only to the sharing of entomological surveillance data and health data collected by the Municipal Health Ministry of Rio de Janeiro, for which access should be granted directly from there via ethical approval. Additionally, all products generated from the mobile phone dataset can only be shared after approval from the provider.

## Appendix 1

See Table 2 and Figs. 7, 8, 9, 10 and 11.

**Table 2:** List of retrieved covariates for the modeling of urban DENV occurrence in the municipality of Rio de Janeiro, including information on the spatial resolution, data source and the required pre-processing step before running zonal statistics on antenna tessellation level.

Category	Subcategory	Proxy	Spatial resolution	Source	Required pre-processing step
Hazard	Egg	Estimated *Aedes* mean egg count per trap (MET)	200 m	Ovitrap	Interpolation^39^
	Larva	Estimated *A. aegypti* mean larva count per trap (MLT)	200 m	Ovitrap	Interpolation^39^
		Estimated *A. albopictus* mean larva count per trap (MLT)	200 m	Ovitrap	Interpolation^39^
	Larva and pupa	Mean *A. aegypti* larva breteau index (BI)	Survey strata	LIRA	Rasterization
		Mean *A. albopictus* larva breteau index (BI)	Survey strata	LIRA	Rasterization
		Mean *A. aegypti* larva house index (HI)	Survey strata	LIRA	Rasterization
		Mean *A. albopictus* larva house index (HI)	Survey strata	LIRA	Rasterization
Vulnerability	Accessibility	Travel time to closest healthcare facility (TMIST)	H3 spatial grid 0.11 km^2^	IPEA	–
		Total number of healthcare facilities (S001)	H3 spatial grid 0.11 km^2^	IPEA	–
		Travel time to closest public school (TMIET)	H3 spatial grid 0.11 km^2^	IPEA	–
		Total number of public schools (E001)	H3 spatial grid 0.11 km^2^	IPEA	–
		Cumulative opportunity measure to access jobs in 60 minutes (CMATT60)	H3 spatial grid 0.11 km^2^	IPEA	–
		Total number of formal jobs (T001)	H3 spatial grid 0.11 km^2^	IPEA	–
	Centrality	OSM road network centrality by averaged travel time in car	200 m	OpenStreetMap	OpenRouteService^44^
		Count of public transport stations	< 1 m	Data.Rio	–
	Demographics	Number of residents between 0 and 5 years old	30 m	Humanitarian data exchange	–
		Number of residents older than 60	30 m	Humanitarian data exchange	–
	Socio-economic	Average household income per capita (R001)	H3 spatial grid 0$.$11 km2	IPEA	–
	Immunity level	DENV occurrence	< 1 m	Municipal health ministry (MRJ)	Spatio-temporal aggregation
Other	Spatial weight	Human movement flows	Antenna tessellation	Mobile phone data	Ping extraction^40^
	Offset	Residential population density	30 m	Humanitarian data exchange	–

## Appendix 2. Significance of vulnerability indicators and spatial eigenvectors

Among the applied vulnerability indicators, the socio-economic variable of average income emerged as the most influential predictor, demonstrating a negative association (Table 3). This suggests that higher average income levels in the municipality of Rio de Janeiro are associated with a reduced risk of dengue infections. Conversely, the indicators of the hypothesized vulnerability categories accessibility and centrality did not exhibit significant predictive power, contradicting our initial assumptions. The same applied to the density of older individuals. However, the density of individuals under five years emerged as a significant predictor in our model, exhibiting a negative estimate, which suggests that a higher concentration of children was linked to fewer dengue cases. This could potentially be explained by the fact that first dengue infection per individual have a higher probability of being clinically mild^70^. Additionally, behavioral factors could play a role, as households with young children may be more vigilant in implementing mosquito control measures, thereby reducing dengue transmission.

**Table 3:** Coefficients, standard errors, and p-values for the proposed vulnerability indicators in the extended QP-GLM, which considers hourly human-mosquito biting risk and spatial eigenvectors in the municipality of Rio de Janeiro. Coefficient estimates and standard errors are reported at the link scale.

Vulnerability Class	Coefficients	$\hat{\beta }$	$\hat{\sigma }_{\hat{\beta }}$	$Pr(>|z|)$
Socio-economic	Average household income	− 6.2719	0.7059	$<2e^{-16}$
Accessibility	Travel time to closest healthcare facility	− 0.1221	0.2665	6.47$e^{-1}$
	Total number of healthcare facilities	− 1.2820	0.5748	2.59$e^{-2}$
	Travel time to closest public school	− 0.3676	0.3162	2$\cdot$45$e^{-1}$
	Total number of public schools	0.1972	0.2977	5.08$e^{-1}$
	Cumulative opportunity measure to access jobs in 60 minutes	− 0.0901	0.2497	7$\cdot$18$e^{-1}$
	Total number of formal jobs	− 4.5421	2.2695	4.55$e^{-2}$
Centrality	Standardized OSM road network centrality by average travel time in car	1.4925	0.6917	3$\cdot$11$e^{-2}$
	Count of public transport stations	0.0893	0.0538	9.72$e^{-2}$
Demographics	Number of residents between 0 and 5 years old	− 3.8328	0.7463	3$\cdot$23$e^{-7}$
	Number of residents older than 60 years	0.1224	0.6864	8.59$e^{-1}$
Immunity level	Estimated level of immunity (2015)	1.3946	0.4481	1.90$e^{-3}$
	Estimated level of immunity (2016)	1.5296	0.4095	1.95$e^{-4}$
	Estimated level of immunity (2017)	0.4041	0.2507	1.07$e^{-1}$
	Estimated level of immunity (2018)	− 0.7807	0.4332	7.18$e^{-2}$
	Estimated level of immunity (2019)	1.7539	0.5135	7.00$e^{-4}$
	Estimated level of immunity (2020)	0.5402	0.2855	5.87$e^{-2}$
	Estimated level of immunity (2021)	2.9644	0.5117	8.61$e^{-9}$

In the assessment of the vulnerability class of immunity, our analysis indicated that the calculated significance values for each year of past infections depend on the magnitude of the outbreak pattern (cf. Fig. 12). The years with minor outbreaks and less spatial variance in DENV occurrence (2017, 2018, 2020) exhibited either no significant association or marginally statistically significant association, whereas the major outbreak years with larger spatial variance in DENV occurrence (2016, 2019) showed lower p-values. The most recent year in our analysis, 2021, yielded the highest p-value among the hypothesized immunological vulnerability indicators, despite the occurrence of lower dengue incidence. Surprisingly, most estimates of this vulnerability category were positive, contrary to our initial hypothesis about past infections conferring population immunity. We hypothesized that this is related to the fact that environmental factors facilitating transmission are overruling marginal gains in population immunity (under the assumption that cross-immunity between subsequent serotypes or genotypes is relevant). The complex immunological interactions between infections with the four dengue serotypes over time are not further discussed in this context^24^. In brief, past infections with a heterologous serotype confer short-term cross-immunity, while past infections with a homotypic serotype confer long-term immunity to the same serotype. The duration and effect size of the heterologous cross-immunity and potentially enhancement is dependent on the time interval between the infections as well as on the specific sequence of the serotypes and their genotypic similarity^52,70^. These complex immunological interactions between dengue serotypes make it challenging to utilize spatial distribution patterns of dengue cases from previous years to model immunity levels. Here we can only show the possible existence of a confounding factor not accounted for in the model but influential in driving the spatial distribution of dengue cases at the sub-neighborhood scale.

It is important to note that the presented results are dependent on the selection and calculation methods for vulnerability indicators and spatial eigenvectors. Not all additional variables showed significance in our model, underscoring the nuanced impact and selective relevance of certain variables within such broader predictive frameworks. The significance of the proposed human-mosquito biting risk indicator did also diminish in a more extended spatial model. The spatial eigenvectors (cf. Fig. 13) effectively absorbed a significant portion of the spatial autocorrelation present in the residuals of the proposed QP-GLM. They can be instrumental in formulating additional hypotheses regarding potential missing covariates or confounding factors for integration into future models. Furthermore, they can serve as a tool for testing spatially varying regression coefficients.
